# Spatiotemporal evolution and influencing factors of urban shrinkage in the Yellow River Basin, China

**DOI:** 10.1371/journal.pone.0264236

**Published:** 2022-03-22

**Authors:** Zhenxing Jin, Chengxin Wang, Shangkun Yu, Shuai Zhang, Xiaoming Ding

**Affiliations:** 1 College of Geography and Environment, Shandong Normal University, Jinan, China; 2 Collaborative Innovation Center of Human-Nature and Green Development in Universities of Shandon, Jinan, China; 3 Communist Party China, Shandong Provincial Party School, Jinan, China; Northeastern University (Shenyang China), CHINA

## Abstract

The phenomenon of urban shrinkage has spread across the world as the political and economic landscape changes worldwide. The urban development in China has entered a post-development era characterized by coexisting urban expansion and local shrinkage whilst urbanization continues to progress in this country. This paper investigates the urban shrinkage of 80 cities in the Yellow River, China from the perspective of population and economy, based on urban shrinkage models drawing from western countries. It also analyzes the spatiotemporal evolution and influencing factors of urban shrinkage in this area using a spatial panel data model. The results show the following. (1) The phenomenon of urban shrinkage in the Yellow River Basin has gradually occurred and the degree of shrinkage has aggravated. (2) The shrinking cities in the Yellow River Basin are mainly population-related and potential. (3) The phenomenon of urban shrinkage in the Yellow River Basin shows significant spatiotemporal differences. Shrinking cities are mainly distributed in the upper and middle reaches of the Yellow River Basin and the number of shrinking cities has gradually increased over time. (4) In terms of influencing factors, financial, traffic, and medical conditions have a greater impact on population-related and economy-related shrinkage in these cities.

## Introduction

The world’s political landscape has remained relatively stable after World War II, producing a peaceful environment for the economic recovery of all countries. However, de-industrialization, financial crisis, and resource depletion have given rise to shrinkage to varying degrees in many cities across the world. This phenomenon occurred mainly in traditionally developed countries such as Britain and Germany at first, and then expanded to some cities in the former socialist countries of Eastern Europe, the “rust belt” of the United States, Australia, and Japan, which tend increasingly to spread globally [1, 2]. Since the reform and opening-up, China’s economy and urbanization have made significant progress, and its urbanization rate increased from 17.9% at the beginning of the reform and opening-up to 63.89% at the end of 2020, with an average annual growth rate of more than 1%, representing a miracle of urban development. Nevertheless, while great achievements have been made, the difference in urbanization has become increasingly prominent, primarily manifested in the continued expansion of most cities and shrinkage in some cities [3–6]. China’s urban shrinkage has become more and more notable in the following context: Its economic development enters a new normal; urbanization has entered the second half of the stage; the growth rate of the economy and urbanization has slowed; and urban development has entered a post-development era [7]. The concept of “shrinking cities” was formally put forward in the “Key Tasks for New Urbanization Construction in 2019” issued by the National Development and Reform Commission in 2019. In 2020, it clearly set the tasks for shrinking cities to lose weight and strengthen their bodies. This suggests that urban shrinkage has become a new challenge facing China’s urbanization in the future.

The concept of “urban shrinkage” or “shrinking cities” originated from Schrumpfende Städte in German. In 1988, German scholars Häußermann et al. proposed “shrinking cities” in an empirical study on the Ruhr region in Germany, which referred to the economic recession and population loss caused by de-industrialization in Germany [8]. In 1998, the term “shrinking cities” was formally used to refer to cities with large population losses, and later it was widely used in urban research [9]. Scholars in related fields have investigated the concept, quantitative identification, types, influencing factors, and coping strategies of urban shrinkage, though there has not been international consensus on the concept of urban shrinkage yet. Despite this, population loss has become a well-recognized connotation and yardstick of urban shrinkage [10, 11]. Further research revealed economic, social, cultural, and spatial stagnation or decline as an important connotation and characterization of urban shrinkage [12–15]. With the rise of urban big data, scholars also began to utilize remote sensing and night-time light datasets in the quantitative identification of shrinking cities [16–19], in addition to socio-economic and spatial indicators such as population size, industrial structure, and building vacancy rate [20–22]. According to the different geographical distribution of the population of shrinking cities, urban shrinkage is classified into “perforated shrinkage” represented by European industrial cities and “doughnut shrinkage” represented by cities in the rust belt of the northeastern United States [21, 23]. The main influencing factors of urban shrinkage in western countries include de-industrialization, suburbanization, population aging, political and economic system changes, etc [2, 24]. There are two types of policies to tackle urban shrinkage: recovery measures represented by “urban renewal” and adaptation measures represented by “smart shrinkage” [25, 26]. Since urbanization in China started late compared with western countries, the research into urban shrinkage also lags behind. In 2011, Huang He introduced the concept of “smart shrinkage” into China, leading to a surge in the research in this field. At present, China’s research in this field mainly focuses on the identification, measurement, influencing factors, and typical areas of urban shrinkage. In terms of measurement, Chinese scholars mostly define urban shrinkage based on traditional socio-economic indicators. In recent years, urban big data have gradually been utilized in its measurement [27–30]. China’s urban shrinkage is affected by its unique urban administrative hierarchy in addition to other influencing factors such as population aging, resource depletion, natural disasters, etc., while de-industrialization and suburbanization have less impact on Chinese cities [31–33]. In terms of typical areas, the Pearl River Delta have more urban problems due to the higher economic development and faster urbanization in this region; the population outflow is prominent in Northeast China because of its special natural conditions and current economic development; and many scholars have also paid attention to the provinces in central China, the Yangtze River Delta, resource-based cities, and traditional industrial bases [34–39].

More and more attention has been paid to the phenomenon of urban shrinkage by scholars both at home and abroad, and some productive theoretical and empirical studies have been carried out in this field. Nonetheless, many issues that need to be further discussed arise as the research continues to deepen. Speaking of conceptual connotation and quantitative measurement, many scholars employ the decline in urban population or urban population density as the main indicator of urban shrinkage and barely pay attention to economic indicators, though the economy, as the lifeblood of a city, may have an important impact on urban development. In terms of influencing factors, previous studies focused on qualitative analysis, with fewer quantitative studies, while the combination of qualitative and quantitative influencing factors will be the focus of future research. As for the selection of research area, most researchers China in China cast their eyes on Northeast China, a typical area of urban shrinkage, while less attention is paid to the Yellow River Basin. The selection of research areas in the future should take national policies into consideration. In recent years, with the advancement of Internet big data and geographic information technology, it has become possible to collect large-scale high-precision data to characterize urban activities. Therefore, using such data to predict and simulate the status quo and outlook of urban shrinkage will become an important direction.

In 2019, ecological protection and high-quality development of the Yellow River Basin became a major national strategy. Cities are the core of regional development, and scientifically measuring the shrinkage of cities in the Yellow River Basin serves as a crucial condition for promoting the high-quality development of this region. This paper measures the urban shrinkage in the study area from 2010 to 2018 from the dimensions of population and economy, taking the population and per capita GDP of the municipal districts as indicators. It also builds an indicator system with environmental quality, financial status, infrastructure, etc., and analyzes the influencing factors of shrinking cities in the Yellow River Basin using a panel regression model, in order to provide scientific advice for the healthy and high-quality development of cities in the basin. The measurement and identification of urban shrinkage in the Yellow River Basin and its influencing factors not only helps to understand the status quo of urban shrinkage in the Yellow River Basin, and offers Chinese empirical evidence for international research in urban shrinkage, but also provides a reference for a new round of urban planning and high-quality urban development in the Yellow River Basin.

## Study area and methods

### Study area

**Fig 1:**
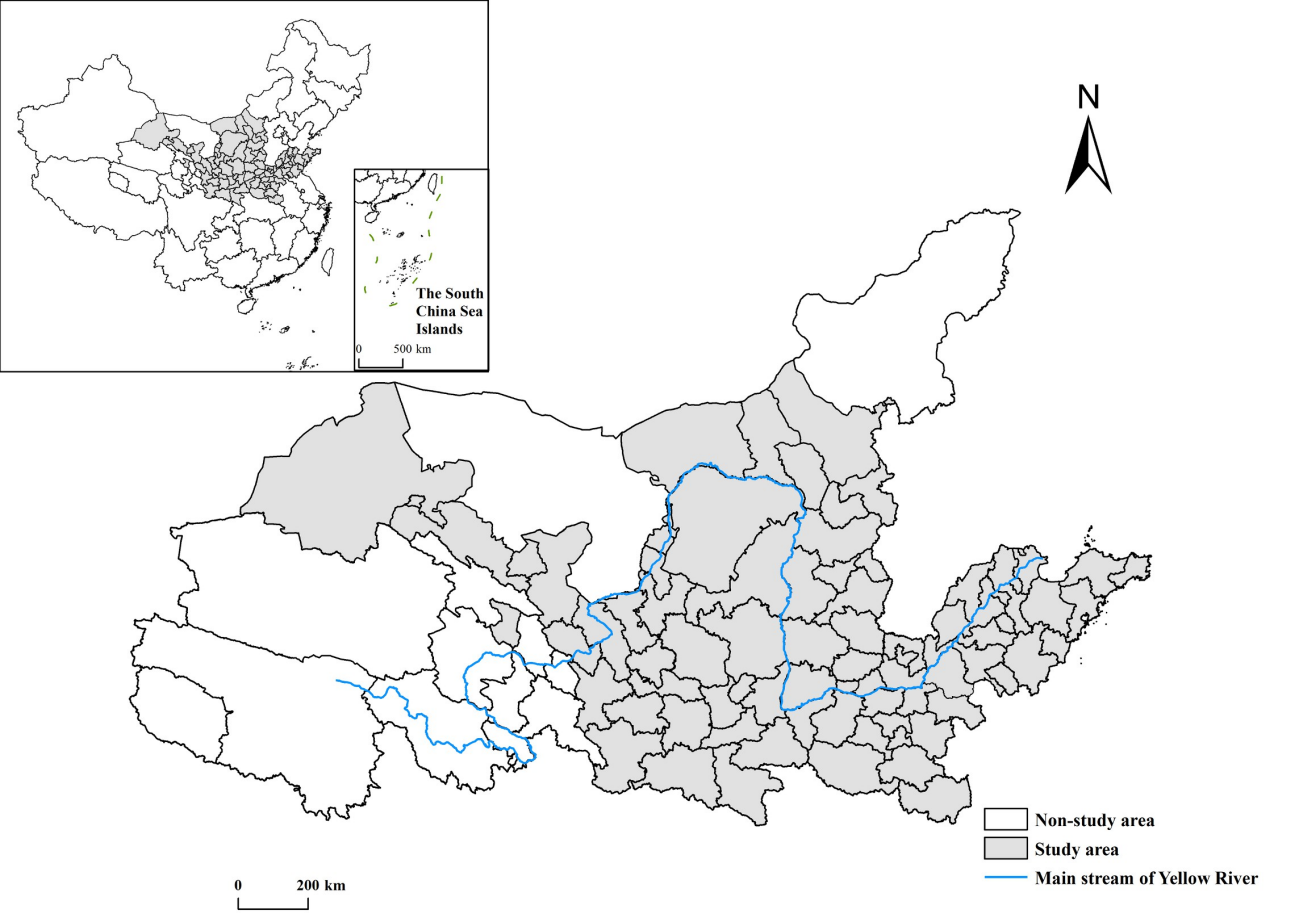
Scope of the study area. The basic map came from a public map from the standard map service website of the Ministry of Natural Resources of China. The drawing approval number is GS (2016)1593.

According to the“Guiding Opinions of the State Council on Promoting Development of the Yangtze River Economic Zone on the Basis of the Golden Waterway of the Yangtze River”, Sichuan Province has been integrated into the Yangtze River Economic Belt as a whole, and the Yellow River only flows through 165 km in Sichuan Province, with a weaker connection with local economic activities. Historically, the eastern part of Inner Mongolia has been more closely connected with northeast China, both economically and socially, and has been included in the spatial scope of the“Northeast Area Revitalization Plan”[40]. On this basis, the scope of the Yellow River Basin defined in this paper includes 8 provinces and autonomous regions except for Sichuan Province and three cities and one league in the eastern Inner Mongolia Autonomous Region. The land area of this basin is about 2.5505 million km^2^, accounting for about 26.57% of China’s total land area. Given data availability, totally 79 prefecture-level cities and 1 provincial county-level city were selected as the study area of this paper (Fig 1).

### Methods

#### Shrinkage model

The shrinkage model is one of the commonly used measurement models in the research of urban shrinkage. It can objectively characterize the increase and decrease of urban population and economic activities. The specific formula is as follows [41, 42]:

$$S_{ip}=(X_{\mathrm{ip}2018}‐X_{\mathrm{ip}2010})/X_{\mathrm{ip}2010}\times 100\%$$ (1)

In the formula, *S*_*ip*_ represents the population-related shrinkage of city *i*. *X*_*ip2018*_ is the urban population of city *i* in 2018, and *X*_*ip2010*_ is the urban population of city *i* in 2010. In addition, economy-related shrinkage is measured using the same method presented above, and the measurement methods of different research periods, such as 2010–2014 and 2014–2018, are also consistent with the measurement of population-related shrinkage.

#### Definition and grading standards of shrinking cities

It is believed in this study that urban shrinkage is a relative concept, and comparisons between cities should be strengthened. Therefore, cities with negative urban permanent population or per capita GDP growth rate are defined as shrinking cities, and cities with positive urban permanent population or per capita GDP growth rate but lower than the average level of cities of the same level are defined as potential shrinking cities [36, 37]. To objectively describe the shrinkage of cities in the study area, the shrinking cities are defined and graded. With reference to the definition and grading standards of shrinking cities, in combination with the actual conditions of the study area, the average annual growth rate of each period is used as a gradient to classify urban shrinkage (S) into 3 categories and 5 grades (Table 1). In particular, potential shrinking cities refer to a city whose population or economic growth rate is lower than the national average level, which means that there is a risk of shrinkage. As the rate of change between the urban population and the per capita GDP of the municipal districts in each period is different, the specific grading standards for each period also differ [43–46]. If a city only shrinks in a single dimension during the same period, it is defined as a single-dimensional shrinking city, namely, a population-shrinking or economically-shrinking city. if a city shrinks in both population and economic dimensions during the same period, it is defined as a two-dimensional shrinking city, that is, a population-economically shrinking city.

**Table 1:** The definition and classification of cities in the Yellow River Basin in population and economic dimensions in different periods (%).

Dimension	Time	Absolute shrinkage		Relative shrinkage	Absolute growth	
		Severe shrinkage	Slight shrinkage	Potential shrinkage	Gentle growth	Rapid growth
**Population dimension**	2010–2014	(-∞,-1.81)	[-1.81,0)	[0,1.81)	[1.81,3.62)	[3.62,∞)
	2014–2018	(-∞,-2.15)	[-2.15,0)	[0,2.15)	[2.15,4.30)	[4.30,∞)
	2010–2018	(-∞,-2.08)	[-2.08,0)	[0,2.08)	[2.08,4.16)	[4.16,∞)
**Economic dimension**	2010–2014	(-∞,-8.25)	[-8.25,0)	[0,8.25)	[8.25,16.50)	[16.50,∞)
	2014–2018	(-∞,-3.36)	[-3.36,0)	[0,3.36)	[3.36,6.72)	[6.72,∞)
	2010–2018	(-∞,-6.50)	[-6.50,0)	[0,6.50)	[6.50,13.00)	[13.00,∞)

#### Panel regression model

Many researchers from other countries attribute the emergence of shrinking cities to de-industrialization, aging, suburbanization, and counter-urbanization. As China’s industrialization and urbanization started late, the factors affecting urban shrinkage are different compared with other countries. Judging from existing studies, the change in people’s opinion of child-bearing, that is, the change in the natural population growth rate affects the population size of a city to a certain extent. Secondly, people often move to cities with good economic development and better infrastructure to pursue a higher quality of life. And the innovation and education levels of a city also become important reasons for people to move in or out. Therefore, with reference to related research [47–50], combined with the actual conditions of the Yellow River Basin, and according to the principles of integrity, systematization, and data availability, a panel regression model is constructed explore the influencing factors of shrinking cities in the Yellow River Basin from 2010 to 2018 and to provide a reference for the future development of cities, taking natural population growth rate (*X*_1_), green coverage area of built-up areas (*X*_2_), local public fiscal revenue (*X*_3_), proportion of science and technology expenditure in fiscal expenditure (*X*_4_), proportion of education expenditure in fiscal expenditure (*X*_5_), road area (*X*_6_), number of certified (assistant) physicians (*X*_7_), and fixed asset investment in the construction of municipal public facilities (*X*_8_) as independent variables from the perspectives of population change, environmental quality, and financial status (Table 2), and urban population size (*Y*_1_) and per capita GDP of the municipal districts (*Y*_2_) as dependent variables.

**Table 2:** The indicator system of the panel regression model of urban shrinkage in the Yellow River Basin.

	Factor layer	Variables	Variable unit
**Dependent variables**	Population shrinkage	Urban population size (*Y*_1_)	10,000 persons
	Economic shrinkage	Per capita GDP of the municipal districts (*Y*_2_)	yuan
**Independent variables**	Population change	Natural population growth rate (*X*_1_)	%
	Environmental quality	Green coverage area of built-up areas (*X*_2_)	hectare
	Financial status	Local public fiscal revenue (*X*_3_)	10,000 yuan
	Technology innovation	Proportion of science and technology expenditure in fiscal expenditure (*X*_4_)	%
	Education conditions	Proportion of education expenditure in fiscal expenditure (*X*_5_)	%
	Traffic conditions	Road area (*X*_6_)	10,000 sq.m
	Medical conditions	Number of certified (assistant) physicians (*X*_7_)	person
	Infrastructure	Fixed asset investment in the construction of municipal public facilities (*X*_8_)	10,000 yuan

### Data source

Considering that urban shrinkage and growth are a gradual process in a long term, the definition of urban shrinkage needs to be based on a certain interval, and it is improper for the period to be too long or too short. In 2010, China’s urbanization rate reached 49.95%, marking an important turning point for the transformation from a rural society to an urban society. Hence, the urban shrinkage in the Yellow River Basin in the three periods of 2010–2014, 2014–2018, and 2010–2018 was measured, with 2010 as the starting point for this study, and 2014 and 2018 as the observation points of urban shrinkage. The specific data were derived from the China Urban Construction Statistical Yearbook, China Urban Statistical Yearbook, and the statistical yearbooks and bulletins of various provinces and autonomous regions from 2010 to 2018.

## Results

The shrinkage model was used to measure the population-related and economy-related shrinkage levels of 80 cities in the Yellow River Basin, and the shrinking cities were classified according to the definition and criteria mentioned above. From the perspective of absolute shrinkage, the absolutely shrinking cities in the Yellow River Basin from 2010 to 2018 were dominated by absolute shrinkage in population, without absolutely shrinking cities in both population and economy. Cities with absolute shrinkage in population were centered on Ordos City, spreading to the south and north, mainly distributed in the middle reaches of the Yellow River Basin. The number of cities with absolute shrinkage in economy was relatively small, mainly distributed in the border between Inner Mongolia and Shanxi Province, namely, Ordos, Shuozhou, and Datong, all resource-based cities. With an aging economic structure, once resources become exhausted in these cities, the loss of population and capital will be more serious, thus necessitating an optimized industrial structure. In terms of relative shrinkage, cities with relative shrinkage in population were more and widely distributed in all provinces except Qinghai Province, especially in the middle reaches of the Yellow River Basin. Cities with relative shrinkage in economy alone or in both population and economy overlapped and were mainly distributed in Henan, Shandong, and other places in the lower reaches of the Yellow River Basin. Areas densely occupied with relatively shrinking cities were mostly surrounded by provincial capitals, sub-provincial cities, and national regional central cities, such as Jinan, Qingdao, Zhengzhou, Luoyang, and Xi’an. These cities had higher urban competitiveness, and affected by the siphonic effect, attracted population, capital, and industries from surrounding areas to gather here, while their marginal cities experienced relatively significant shrinkage due to the shadow effect. From the perspective of its evolution, the number of cities with absolute shrinkage in population or economy in the Yellow River Basin gradually increased, and cities with absolute shrinkage in both population and economy also emerged, indicating that the phenomenon of urban shrinkage in the Yellow River Basin began to occur and spread.

### Classification of urban shrinkage in the Yellow River Basin

To further identify the degree of urban shrinkage and provide more targeted countermeasures and suggestions for future urban development, the shrinking cities were classified as per the criteria presented in Table 1 (Tables 3 and 4).

**Table 3:** Hierarchical statistics of shrinking cities in the Yellow River Basin from 2010 to 2018.

Items	Potential shrinkage		Slight shrinkage		Severe shrinkage	
	quantity/pcs	proportion/%	quantity/pcs	proportion/%	quantity/pcs	proportion/%
**Population-related shrinkage**	40	50.00	5	6.25	2	2.50
**Economy-related shrinkage**	29	36.25	3	3.75	0	0.00
**Population and economy-related shrinkage**	16	20.00	0	0.00	0	0.00

**Table 4:** Hierarchical statistics of shrinking cities in the Yellow River Basin in 2010–2014 and 2014–2018.

Time	Items	Potential shrinkage		Slight shrinkage		Severe shrinkage	
		quantity/pcs	proportion/%	quantity/pcs	proportion/%	quantity/pcs	proportion/%
**2010–2014**	**Population-related shrinkage**	35	43.75	6	7.50	2	2.50
	**Economy-related shrinkage**	34	42.50	4	5.00	0	0.00
	**Population and economy-related shrinkage**	15	18.75	0	0.00	0	0.00
**2014–2018**	**Population-related shrinkage**	37	46.25	10	12.50	5	6.25
	**Economy-related shrinkage**	14	17.50	4	5.00	6	7.50
	**Population and economy-related shrinkage**	7	8.75	3	3.75	1	1.25

There were 7 cities with population-related shrinkage in the Yellow River Basin from 2010 to 2018, accounting for 8.75%. Among them, there were 5 slightly shrinking cities, including Shizuishan, Zhongwei, and Baotou, 2 severely shrinking cities, namely, Ordos and Jiyuan, and 40 potential shrinking cities, accounting for 50.00%. There were 3 economically- shrinking cities, accounting for 3.75%. Among them, there were 3 slightly shrinking cities, namely, Ordos, Datong, and Shuozhou, 0 severely shrinking cities, and 29 potential shrinking cities, including Jiayuguan, Jinchang, Baiyin, etc., accounting for 36.25% of the number of economically-shrinking cities. There were no shrinking cities in both population and economy, but there were 16 potential shrinking cities, accounting for 20.00% of all the cities being studied, including Jinchang, Baiyin, and Jiuquan. Generally, the urban shrinkage in the Yellow River Basin during the research period was dominated by potential shrinking cities and mainly represented by the shrinkage of the population in the municipal districts. This shows that the urban development of the Yellow River Basin is in the midst of growth as a whole, but the population and economic growth rate are lower than the average level nationwide, with insufficient development momentum. The growth rate of urban population and per capita GDP in many cities was lower than the national average, especially in terms of population. Since most cities in the Yellow River Basin are located between the Beijing-Tianjin-Hebei urban agglomeration and Yangtze River Delta urban agglomeration, some cities in the Yellow River Basin were not attractive enough compared with urban agglomerations, thus resulting in population outflow. The number of cities with absolute shrinkage was relatively small, mainly characterized by shrinkage either in population or economy, and there were no cities with absolute shrinkage in both population and economy, demonstrating that the Yellow River Basin is currently in the initial stage of urban shrinkage, and the phenomenon of shrinkage is not yet significant.

The urban shrinkage in the two periods of 2010–2014 and 2014–2018 was mainly dominated by potential shrinking cities and characterized by population-related shrinkage. The total number of cities with population-related shrinkage rose from 8 in 2010–2014 to 15 in 2014–2018. The total number of economically-shrinking cities increased from 4 in 2010–2014 to 10 in 2014–2018, with a larger scope and severely shrinking cities emerging. Notably, the number of potential shrinking cities dropped significantly by 20. The total number of shrinking cities in both population and economy in 2014–2018 increased from 0 in 2010–2014 to 4 in 2014–2018. In 2010–2014, the cities with shrinkage in both population and economy shrank potentially. In 2014–2018, although the total number of such cities decreased, cities with slight shrinkage and severe shrinkage began to appear. In general, the urban shrinkage within the study area was mainly population-related, and population-related shrinkage occurred earlier than economy-related shrinkage. There were fewer shrinking cities in both population and economy. Potential shrinking cities accounted for the majority of shrinking cities, while the proportion of absolutely shrinking cities was relatively small. The degree of shrinkage tended to aggravate gradually, which should draw close attention.

Through the definition and classification of shrinking cities in the Yellow River Basin during the three periods of 2010–2018, 2010–2014, and 2014–2018, it was found that the shrinking cities were mainly population-related, and potential shrinkage was the dominant form whether in shrinking cities in population, economy, or both. The proportion of slightly and severely shrinking cities was relatively small.

### Spatiotemporal evolution of shrinking cities in the Yellow River Basin

**Fig 2:**
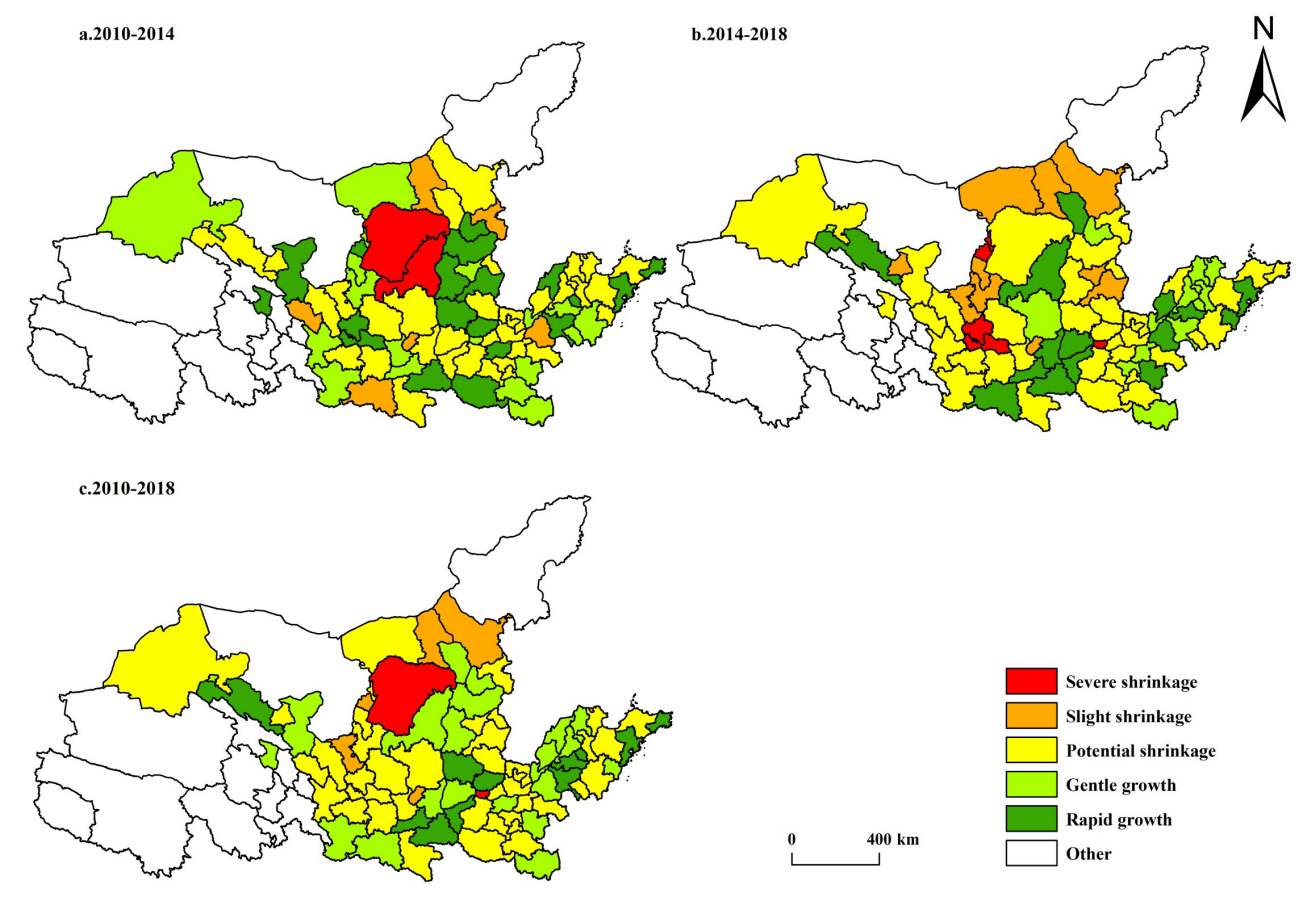
Spatiotemporal differentiation of population-shrinking cities in the Yellow River Basin. The basic map came from a public map from the standard map service website of the Ministry of Natural Resources of China. The drawing approval number is GS (2016)1593.

In terms of the spatiotemporal evolution of population-shrinking cities in the Yellow River Basin (Fig 2), the population-shrinking cities in the Yellow River Basin from 2010 to 2018 were mainly distributed in central and southern Inner Mongolia, Ningxia, Shaanxi, and Henan provinces, showing a “point-axis” pattern. Shandong Province, the economic leader in the Yellow River Basin, did not experience population-related shrinkage, indicating that a strong economic foundation remains an important factor in attracting population inflows. According to the National Sustainable Development Plan for Resource-Based Cities (2013–2020) issued by the State Council, Ordos, Shizuishan, Baotou, and Tongchuan are all resource-based cities, and all of these cities except Ordos have become resource-exhausted cities. Apparently, for resource-based cities, the depletion of resources seriously hinders the development of the local economy, leading to an increase in unemployment and even population loss. There were a large number of potential shrinking cities widely distributed in Gansu, Ningxia, Shaanxi, Henan, and other provinces. Local governments should pay attention to it and take appropriate measures to reduce the risk of transformation from potential shrinking cities to shrinking ones. By comparing the population-shrinking cities in 2010–2014 and 2014–2018, it was easy to find that the geographical distribution of shrinking cities gradually became decentralized from centralization. For example, the shrinking cities in 2010–2014 were mainly distributed at the junction of Shanxi, Shaanxi, and Inner Mongolia, while in 2014–2018, such cities were widely distributed in Ningxia, Inner Mongolia, and Henan provinces, with an increase in both number and severity. The number of slightly and severely shrinking cities increased by 4 and 3, respectively.

**Fig 3:**
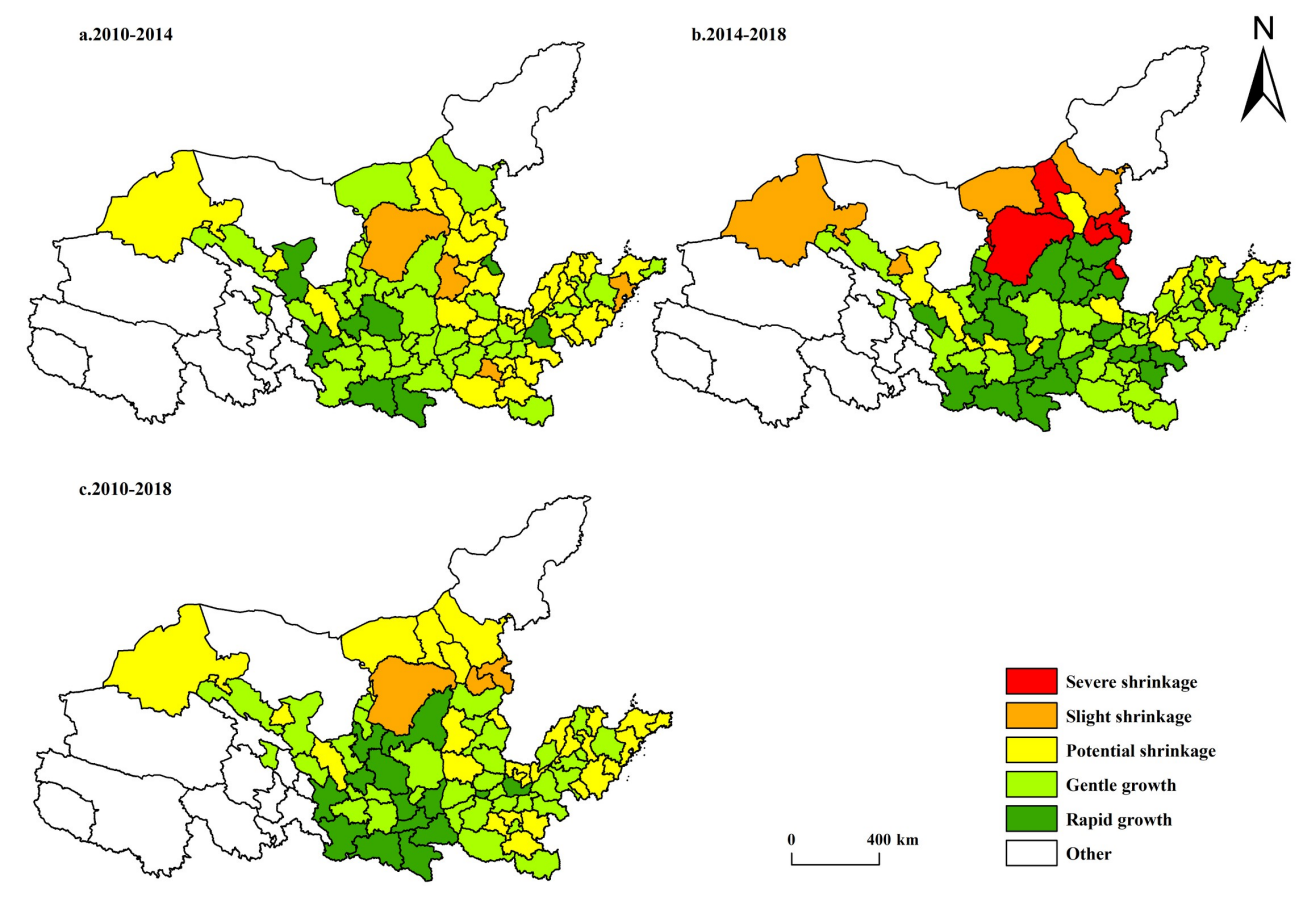
Spatiotemporal differentiation of economically-shrinking cities in the Yellow River Basin. The basic map came from a public map from the standard map service website of the Ministry of Natural Resources of China. The drawing approval number is GS (2016)1593.

From the perspective of the spatiotemporal evolution of economically-shrinking cities in the Yellow River Basin (Fig 3), there were few economically-shrinking cities in the Yellow River Basin from 2010 to 2018, characterized by slight shrinkage in Ordos, Datong, and Shuozhou, mainly distributed in clumps at the junction of Inner Mongolia and Shanxi provinces. Judging from the nature of economically-shrinking cities, these three cities are resource-based cities with a single and aging industrial structure. Industrial production around resource exploitation is the main impetus for local economic development. For example, the proportion of tertiary industries in Ordos in 2010 was 2.7:60.2:37.1. Although its industrial structure was optimized to 3.1:52.3:44.6 by 2018, it still lagged behind the national average of 7.0:39.7:53.3 in the same year. In other words, the industrial structure of this city needed to be optimized as soon as possible. From 2010 to 2014, economically-shrinking cities were distributed in the eastern, central, and western parts of China, but the degree and number of shrinking cities in central and western China were higher than those in the eastern region. From 2014 to 2018, the economically-shrinking cities in the Yellow River Basin experienced eruptive growth, with a significant increase both in number and extent, but they were still mainly distributed in the central and western regions. By comparing the economically-shrinking cities in 2010–2014 and 2014–2018, it was easy to find that the geographical distribution of shrinking cities gradually concentrated to the north of the mainstream of the Yellow River, mainly distributed at the junction of Inner Mongolia, Shaanxi, Shanxi, and other provinces. Moreover, the number and severity of shrinking cities showed an obvious upward trend, and the number of severely shrinking cities greatly increased and all of them were resource-based cities.

**Fig 4:**
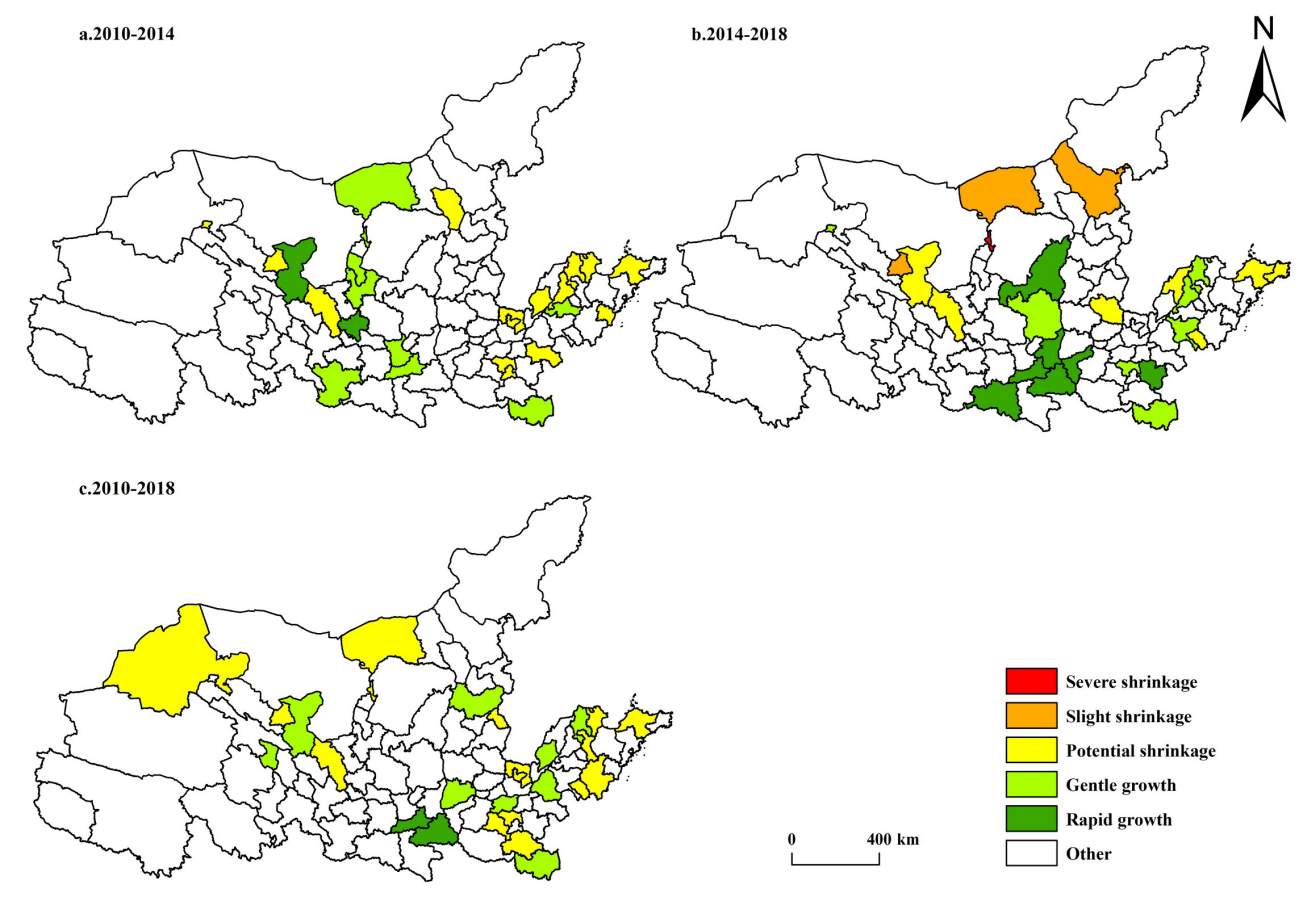
Spatiotemporal differentiation of shrinking cities in both population and economy in the Yellow River Basin. The basic map came from a public map from the standard map service website of the Ministry of Natural Resources of China. The drawing approval number is GS (2016)1593.

Speaking of the spatiotemporal evolution of shrinking cities in both population and economy in the Yellow River Basin (Fig 4), from 2010 to 2018, there were no cities with both population-related and economy-related shrinkage in the Yellow River Basin, but there were many potential shrinking cities widely distributed in Shandong, Henan and other places and mainly around the provincial capitals Jinan, Zhengzhou, the sub-provincial city Qingdao, and the national regional center Luoyang. Cities with higher administrative levels often enjoy better infrastructure and stronger resource control capabilities. Compared to surrounding cities, the competitiveness of these cities is more superior. Therefore, they are more likely to attract the population and industries of the surrounding cities to gather here. Affected by the siphonic effect between cities, there were more potential shrinking cities surrounding these cities. Jinchang, Wuhai, Tongchuan, Yangquan, and other cities are mature or declining resource-based cities and their economic development around resource exploitation was facing bottlenecks. And their population and economic growth rates were lower than the national average, contributing to a risk of shrinkage. By comparing the shrinking cities in both population and economy in 2010–2014 and 2014–2018, it was easy to find that the shrinking cities in both population and economy in the Yellow River Basin emerged and tended to aggravate. In 2014–2018, such cities were mainly distributed in Inner Mongolia and Gansu provinces. It could be seen that most cities in the Yellow River Basin were still dominated by growth, but the phenomenon of urban shrinkage began to appear, and the number and severity of shrinking cities continued to increase over time with changes in the development environment.

### Influencing factors of shrinking cities in the Yellow River Basin

The phenomenon of urban shrinkage is a result of multiple factors, and its influencing factors differ significantly according to the nature, function, and spatial distribution of the city. Descriptive statistics were conducted based on the indicator system of the panel regression model of urban shrinkage in the Yellow River Basin (Table 5) to understand the data more directly. To ensure the validity of the panel regression results and prevent false regression in the data, LLC and ADF were used to test the stability of the research data, and the results demonstrated that they were stable.

**Table 5:** Descriptive statistics of variables.

Variable	Unit	Mean	Std. Dev.	Min	Max
***Y***_**1**_	10,000 persons	80.11676	79.71007	11.6	576.56
***Y***_**2**_	yuan	57362.95	38003.39	6222	326030
***X***_**1**_	%	6.691083	4.573366	-15.9	40.6
***X***_**2**_	hectare	4414.108	4499.151	12	39229
***X***_**3**_	10,000 yuan	760416.5	1272731	13765	10261393
***X***_**4**_	%	1.365761	1.271945	0.0132189	18.16803
***X***_**5**_	%	18.44062	6.893093	4.93176	149.1362
***X***_**6**_	10,000 sq.m	1564.95	1663.616	52	10601
***X***_**7**_	person	4404.436	4859.391	310	32177
***X***_**8**_	10,000 yuan	328952.6	614722	2462	5032424

A panel regression analysis was performed on the influencing factors of urban population-related and economy-related shrinkage in the Yellow River Basin from 2010 to 2018. To weaken heteroscedasticity and avoid the influence of excessive abnormal fluctuations of data on the results, logarithm processing was performed on the raw data, and fixed effects, random effects, and system GMM models were used for regression analysis on the data (Table 6). According to the Hausman test and the calculation results of various models, it was found that the explanatory power of the fixed-effect model was better than other models. Therefore, it was utilized to explore the influencing factors of urban population-related and economy-related shrinkage [51, 52].

**Table 6:** Panel regression results of factors affecting urban shrinkage in the Yellow River Basin.

Explained variable	*Y*_1_			*Y*_2_		
	Fixed effect	Random effect	System GMM	Fixed effect	Random effect	System GMM
ln*X*_1_	0.0089 (0.67)	0.0135 (0.81)	0.0110 (0.70)	0.0252 (1.06)	0.0195 (-0.79)	-0.0032 (-0.16)
ln*X*_2_	0.0383*** (3.65)	0.0753*** (5.87)	0.0067 (0.75)	0.0340* (1.80)	0.0303 (-1.61)	0.0080 (0.20)
ln*X*_3_	0.0983*** (8.18)	0.1470*** (10.33)	0.0056 (0.21)	0.2405*** (11.04)	0.2332*** (-11.03)	0.1298* (1.88)
ln*X*_4_	-0.0127* (1.85)	0.0033(0.38)	-0.0112 (-1.24)	0.0144 (1.15)	0.011 (-0.87)	0.0129 (0.71)
ln*X*_5_	0.0403* (1.92)	0.0452* (1.74)	-0.0092 (-0.47)	0.0403 (0.96)	0.0285 (-0.67)	-0.0388 (-0.58)
ln*X*_6_	0.1751***(11.62)	0.1218*** (8.59)	0.0053 (0.30)	0.2862*** (11.15)	0.2577*** (8.83)	0.0914** (2.43)
ln*X*_7_	0.1200*** (7.22)	0.2213*** (11.20)	0.0326 (1.03)	0.2393*** (7.92)	0.1702*** (-5.78)	0.0599 (0.98)
ln*X*_8_	0.0197*** (3.62)	0.0353*** (5.22)	0.0011 (0.17)	0.0132 (1.34)	0.0092 (-0.92)	0.0066 (0.52)
*R* ^*2*^	0.3287	0.3215		0.4892	0.4842	
F-statistics	38.68			74.71		

Notes:*, ** and *** indicate significance at 10%, 5%, and 1% confidence intervals, respectively.

The regression results revealed that a total of 7 indicators had an impact on the increase or decrease of the urban population size. According to the correlation coefficient of the influencing factors, the degree of impact of the indicators was *X*_6_, *X*_7_, *X*_3_, *X*_5_, *X*_2_, *X*_8_, and *X*_4_ in descending order. Among them, the green coverage area of built-up areas (*X*_2_), local public fiscal revenue (*X*_3_), road area (*X*_6_), number of certified (assistant) physicians (*X*_7_), and fixed asset investment in the construction of municipal public facilities (*X*_8_) passed the 1% confidence interval test, suggesting that they had a significant positive effect on the change of population size. From the perspective of economy-related shrinkage, totally 4 indicators had an impact on the increase or decrease of the city’s economic scale. The degree of impact of these indicators was *X*_6_, *X*_3_, *X*_7_, and *X*_2_ in descending order. Among them, local public fiscal revenue (*X*_3_), road area (*X*_6_), and the number of certified (assistant) physicians (*X*_7_) passed the 1% confidence interval test, suggesting that they had a significant positive effect on the per capita GDP. Taken together, road area, that is, traffic conditions are the most important factor affecting urban population-related and economy-related shrinkage.

## Discussion

Cities are the epitome of high-quality development in a region. As ecological protection and high-quality development of the Yellow River Basin became a major national strategy, it is of great practical significance to examine the urban shrinkage and influencing factors in the Yellow River Basin. This paper analyzes the spatiotemporal difference and influencing factors of urban shrinkage in the Yellow River Basin based on existing studies and empirical results, combined with the actual conditions of the Yellow River Basin.

### Spatiotemporal differentiation of shrinking cities in the Yellow River Basin

As resource-based cities face the depletion of resources and decline of traditional industries, some cities in the Yellow River Basin have shown signs of population outflow, economic stagnation, and even recession, and urban shrinkage has initially appeared [27]. The number of shrinking cities in the Yellow River Basin has gradually increased over time, with an increasingly worsening trend of shrinkage, and shrinkage has gradually become multidimensional. The shrinking cities were mainly distributed at the junction of Inner Mongolia, Shaanxi, and Shanxi provinces, and the potential shrinking cities were mostly distributed around the regional centers. The formation of shrinking cities in the Yellow River Basin is not unrelated to its special development background and conditions. First of all, the Yellow River Basin has a poorer development foundation compared with the Yangtze and Pearl River basins. The Yellow River basin spans the three topographical steps of China, from west to east across the Qinghai-Tibet Plateau, the Inner Mongolia Plateau, the Loess Plateau, and the alluvial plains of the lower Yellow River. The headwaters of the western Yellow River Basin have an average altitude of more than 4000 m, profuse with glacial landforms; the central area is between 1000–2000 m above sea level, with extensive loess landforms and serious soil erosion; and the eastern area has many alluvial plains and the river courses are higher than the ground, which is susceptible to floods. In general, the ecological environment of the Yellow River Basin is extremely fragile [27, 53]. Secondly, the Yellow River Basin has abundant minerals and energy resources and is known as the “energy basin”. Many resource-based cities with a relatively simple industrial structure are located here, such as Baiyin, Shizuishan, Wuhai, Baotou, Ordos, etc. The job opportunities provided by resource-intensive industries are limited and single, and with the over-exploitation of resources, many resources are on the verge of exhaustion, resulting in economic decline and population loss [39, 54]. Moreover, the Yellow River Basin lacks central cities with strong radiant driving forces. Cities such as Jinan, Hohhot, and Zhengzhou have low primacy ratios, while cities such as Xining, Yinchuan, and Taiyuan have relatively high primacy ratios, but their economic strength is weak with limited radiant driving forces, and their attraction to the population in the basin is insufficient, which indirectly causes the population in the basin outflows to the surrounding Yangtze River Delta, Beijing-Tianjin-Hebei and other urban agglomerations, thereby intensifying the urban shrinkage in the basin.

### Analysis on influencing factors of shrinking cities in the Yellow River Basin

The imbalance of regional economic development is an important cause of population flow between regions. Regions with a higher level of economic development usually provide more development opportunities and jobs and thus attract more population inflows [41, 44]. With the development of the economy and society and the enrichment of material life, people’s pursuit of a better life is no longer limited to higher incomes, and more attention is paid to a better ecological environment and convenient infrastructure. Therefore, urban greening, medical and health care, and infrastructure conditions have gradually become important factors in attracting population inflows. According to the regression results, the correlation coefficient of road area to the urban population size of the Yellow River Basin was 0.1751, which was the largest among the influencing factors. This indicates that urban road area, namely, traffic conditions, the degree of traffic convenience, has an important impact on the increase or decrease of the urban population size of the Yellow River Basin. The analysis of statistical data demonstrates that the cities in the lower reaches of the Yellow River Basin have better greening, transportation, medical, and health care conditions compared with those in the upper and middle reaches. As a result, the cities with population-related shrinkage are mostly distributed in the upper and middle reaches. In addition, the proportion of science and technology expenditure and education expenditure in fiscal expenditure also passed the 10% confidence interval test, suggesting that the development of science and technology and the optimization of educational resources will promote regional population growth and curb population loss.

The level of local public fiscal revenue is an important manifestation of the quality of a city’s economic development, and higher fiscal revenue provides a guarantee for the development of the local economy. The two complement and promote each other [4]. Perfect transportation facilities not only facilitate the flow of people but also promote mutual exchanges between industries as well as the growth of the local economy. Conversely, congested traffic conditions hinder the exchanges between people and businesses and are likely to cause economic slowdown, stagnation, or even shrinkage. The sound medical conditions provide sufficient healthy labor for local economic development and vitalize the development of the city, and effectively prevent the economy-related shrinkage of the cities in the Yellow River basin while ensuring the health of urban residents. This shows that the population size and economic scale of a city interact with each other. The higher the degree of urban economic development, the more development opportunities it can provide, and the more population it can attract. The continuous inflow of population will provide a sufficient labor force for the further development of the urban economy.

In addition, the differences in natural background conditions, the depletion of urban resources, and the siphonic effect between cities also serve as important reasons for the shrinkage of some cities [27, 39, 55]. The middle and upper reaches of the Yellow River Basin, especially the upper reaches, are dominated by a continental climate. The climate, water resources, traffic conditions, and industrial foundations here are poorer compared with the lower reaches. And there are many mountains and rivers in this area. Therefore, its economic attractiveness is weaker than that of downstream regions. For resource-based cities that rely on non-renewable energy sources such as coal and petroleum as their economic pillars, over-exploitation and consumption of resources will have a great impact on the development of the city. The exhaustion of resources will lead to shutdowns of many factories, unemployment, and economic stagnation. For example, resource-based cities such as Ordos, Jinchang, Wuhai, Tongchuan, and Yangquan are in bad need of industrial restructuring to alleviate their population outflow and economic slowdown. The siphonic effect between cities means that if the central city and the surrounding cities in a certain area do not have a higher level of coordinated development, the central city has higher competitiveness and is more likely to attract the population and economic resources of the surrounding cities [55]. This siphonic effect poses a potential risk of turning the surrounding cities into shrinking cities, and especially, the construction of high-speed railways improves the traffic conditions between cities and exacerbates the siphonic effect [56]. For example, Jinan (the capital city of Shandong Province) and Zhengzhou (the capital city of Henan Province), the national central city Luoyang, and the sub-provincial city Qingdao boast sound infrastructure conditions and stronger resource control capabilities, while many of their surrounding cities face potential risks of shrinkage.

## Conclusion

Cities will not expand and grow forever since the phenomenon of shrinkage already exists. Growth and shrinkage are two opposite and unified aspects of urban development and civilization. Urban shrinkage is a form of urban transformation and should be treated rationally. It can only be “coped with” but not “solved” radically [7]. This paper systematically assesses the urban shrinkage of the Yellow River Basin from 2010 to 2018 with prefecture-level cities as the research unit and explores its spatiotemporal evolution and influencing factors. The definition and grading of shrinking cities in the Yellow River Basin at various periods demonstrate that the phenomenon of urban shrinkage in the Yellow River Basin has gradually emerged and is dominated by population-related shrinkage. Relative shrinkage is its main manifestation, while absolutely shrinking cities account for only a small proportion. The urban shrinkage in the Yellow River Basin is dominated by one-dimensional shrinkage in population or economy. Although cities with shrinkage in both population and economy have appeared, the number of such cities is small. The phenomenon of urban shrinkage in the Yellow River Basin has significant spatiotemporal differences. Temporally, the number of shrinking cities in the Yellow River Basin has gradually increased, and the degree of shrinkage tends to aggravate. Spatially, the shrinking cities in the upper and middle reaches of the Yellow River Basin are more and severe, while shrinking cities in the downstream areas are less and mild. Financial, traffic, and medical conditions are the common influencing factors of population-related and economy-related shrinkage. Environmental quality and infrastructure also have a great impact on changes in urban population size. Furthermore, differences in natural background conditions, depletion of resources, and the siphonic effect between cities also represent important influencing factors of urban shrinkage.

Shrinking cities in the Yellow River Basin have continued to increase and worsen over time, which has hindered their high-quality development to a certain extent, which requires close attention from local authorities. Urban shrinkage has significant differences in spatial distribution, and the influencing factors of different shrinking cities are also significantly heterogeneous. For relatively shrinking cities, since their population loss and economic recession are not significant, efforts should be made to vigorously develop competitive industries, improve urban infrastructure, and provide local residents with a better and more convenient living environment, thus increasing their urban attractiveness. For absolutely shrinking cities characterized with significant population loss, economic decline, and obvious shrinkage, it is necessary to accelerate their industrial upgrading, attract domestic and foreign high-tech and emerging industries to settle in, and create new economic growth points, thereby realizing urban rejuvenation. Different measures are required to cope with different types of shrinkage, but a series of measures to reverse the phenomenon of shrinkage is not the only way out for shrinking cities. Facing shrinkage directly and making “smart shrinkage” plans to improve the comfort of urban residents and build a happy small city is also an important choice for the urban development of shrinking cities. In short, this study explores the law of urban shrinkage in the Yellow River Basin from the dimensions of population and economy, which by and large enriches the connotation of theories related to urban shrinkage. Urban shrinkage is classified into 3 categories and 5 grades in comparison with the national average annual growth rate of the population during the same period, and this study analyzes the urban shrinkage in the Yellow River Basin from the perspective of absolute shrinkage and relative shrinkage and proposes the concept of potential shrinking cities, which can deepen the understanding of urban shrinkage and change people’s negative perception of shrinking cities [37].

There are several limitations to this study. Due to the availability of data, this paper only investigates 80 cities in the Yellow River Basin. In future studies, attention should be paid to the comprehensive utilization of multiple data, especially night-time light datasets. The study is performed only on prefecture-level cities, without involving county-level cities or smaller scales, and the research scale may be reduced to county-level cities, towns, or streets in the future to reveal the law of urban shrinkage from a more microscopic perspective. Shrinking cities are comprehensively affected by multiple factors, and their evaluation indicator system needs to be continuously improved in specific research and practice in the future, and follow-up research on the influencing factors of shrinking cities should continue, in order to provide more valuable references for high-quality development of the Yellow River Basin.

## Supporting information

S1 Data (XLSX)
